# Impact of Remote Patient Monitoring On Major Depressive episode And Bipolar Disorder Treatment Outcomes: EC-102 Randomized Controlled Trial

**DOI:** 10.1192/j.eurpsy.2026.12057

**Published:** 2026-07-31

**Authors:** A. Yrondi, A. Bourla, R. Belzeaux, E. Olié, R. David, J. Mallet, A. Amad, A. Sauvaget, M. Polosan, J. Holtzmann, F. Chevrier, E. Haffen, D. Bennabi, F. Stéphan, E. Artiges, A. Amrandi-Chickh, S. Bulteau, W. El-Hage, V. Rousseau, N. Lele, C. Martelli, L. Mekaoui, D. Jacon, G. Chabridon, L. Dormegny Jeanjean, F. Hermelin, E. Touré Cuq, P.-M. Llorca

**Affiliations:** 1 Service de Psychiatrie et de Psychologie Médicale de l’adulte, Centre Expert Dépression Résistante FondaMental, CHU de Toulouse, Hôpital Purpan, ToNIC Toulouse NeuroImaging Centre, Université de Toulouse, INSERM, UPS, Toulouse; 2 Department of Psychiatry, Saint-Antoine Hospital, Sorbonne University; 3 Assistance publique Hôpitaux de Pari; 4 Medical Strategy and Innovation Department, Clariane; 5 NeuroStim Psychiatry Practice, Paris; 6 IGF, Univ. Montpellier, CNRS, INSERM; 7 Service de psychiatrie; 8 Service de Psychiatrie d’Urgence et de Soins Aigus, CHU Montpellier; 9 Université Montpellier; 10 IGF, CNRS, INSERM, Montpellier; 11 CHU de Nice; 12 Université Côte d’Azur, Nice; 13 Service de psychiatrie adulte, Hôpital Louis Mourier, AP- HP, Paris; 14 Service de psychiatrie adulte, EPSM du Loiret, Centre Hospitalier Universitaire d’Orléans; 15 B-CLINE, Laboratoire Interdisciplinaire pour l’Innovation et la Recherche en Santé d’Orléans (LI²RSO), Université d’Orléans, Orléans; 16 U1172 - LilNCog - Lille Neuroscience & Cognition, F-59000, CHU Lille, Lille; 17 EPSM de Vendée, Centre Hospitalier Georges Mazurelle, La-Roche-sur-Yon; 18 Inserm U1216, Univ. Grenoble Alpes; 19 Grenoble Institut de Neurosciences, CHU de Grenoble; 20 Service de Psychiatrie Adulte, CHU Grenoble Alpes, Grenoble; 21 unité de réhabilitation psychosociale, Clinique château Caradoc, Bayonne; 22 Service de Psychiatrie Clinique, CHU de Besançon; 23 CIC-1431 INSERM, Université de Franche-Comté UMR 1322 LINC, Besançon; 24 Service Hospitalo-Universitaire de Psychiatrie Générale et de Réhabilitation Psycho-Sociale 29G01/29G02, CHRU de Brest, Brest; 25 LaB-D Psy, EPS Barthélémy Durand, Etampes; 26 Pôle Hospitalo-Universitaire de Psychiatrie, CHU de Nantes; 27 Inserm U1246 SPHERE, Nantes Université, Nantes; 28 Service de Psychiatrie, CHRU de Tours; 29 Inserm U1253, Université de Tours, Tours; 30 Centre hospitalier la chartreuse, Dijon; 31 Clinique d’Yveline, Vieille-Église-en-Yvelines; 32 Département de Psychiatrie et Addictologie, AP-HP Hôpital Paul-Brousse, Villejuif; 33 GHU Paris Psychiatrie & Neurosciences, CMME, Hôpital Sainte-Anne, Paris; 34 Cabinet de Psychiatrie, Biarritz; 35 Service de psychiatrie adulte, CHU Dijon-Bourgogne, Dijon; 36 Centre de Neuromodulation Non-invasive de Strasbourg (CEMNIS), Service de Physiologie et d’Explorations Fonctionnelles, Hôpitaux Universitaires de Strasbourg; 37 UMR CNRS 7357 ICube, Université de Strasbourg, Strasbourg; 38 Service de psychiatrie, CHU d’Orléans, Hopital de la source, Orléans; 39 Medical department, Resilience, Biarritz; 40 Service de Psychiatrie, CHU de Clermont-Ferrand; 41 Université Clermont-Ferrand , Clermont-Ferrand , France

## Abstract

**Introduction:**

Major depressive disorder (MDD) and bipolar disorder (BD) are chronic, disabling conditions marked by recurrent mood episodes that impair functioning and quality of life. Despite available treatments, remission rates are low, relapse is common, and symptom monitoring between visits remains limited. Remote patient monitoring (RPM) may help by enabling early detection and timely intervention. Edra PRO is a digital medical device indicated in the MDD and BD patient monitoring that collects electronic patient-reported outcomes (ePROs) and sends alerts to healthcare professionals to monitor symptoms remotely. While early data support its feasibility, robust clinical evidence is needed to demonstrate its impact and support wider adoption.

**Objectives:**

The EC-102 trial aims to evaluate the clinical and organisational benefit of Edra PRO compared to usual care, in improving treatment response in adult patients undergoing pharmacological treatment for a moderate to severe major depressive episode, with or without BD, by comparing the impact of Edra PRO versus usual care on response rate, as measured by the MADRS score, over a 6-month period.

**Methods:**

EC-102 is a multicentric, randomized, controlled, single-blind trial comparing Edra PRO to usual care over 6 months. Outcome assessors for the primary endpoint will be blinded to treatment allocation. An interim analysis is planned at 3 months. A total of 594 adult patients diagnosed with a moderate to severe MDD or BD, newly treated or adjusted on treatment, will be enrolled. At least 20% will have BD. Participants will be randomized 1:1 to receive either standard care and use the Edra PRO digital medical device (intervention) or standard care alone (control). The study follows a hybrid design, with one in-clinic visit and the rest conducted remotely through online questionnaires. Patients in the intervention group will complete weekly ePROs on mood symptoms, side effects, and treatment adherence via Edra PRO. Based on predefined clinical thresholds, the system automatically generates alerts that are sent to case managers for review and follow-up. Edra PRO aims to support timely clinical decisions without altering the underlying treatment plan. Usual care will remain unchanged in both groups. The primary endpoint is the difference in 6-month response rates (≥50% reduction in MADRS score from baseline). Secondary endpoints include 3 and 6-month response, remission and relapse rates, quality of life using EQ-5D-5L and SF-36, healthcare use, adherence to treatment, and safety.

**Results:**

First results are expected for late 2026 and will be presented at the next EPA meeting.

**Image 1:**
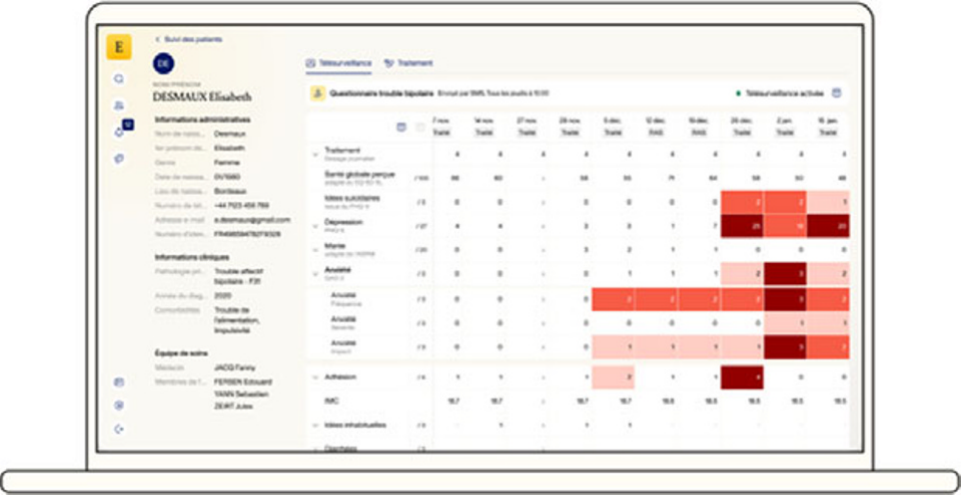
Image 1: Long description.

**Image 2:**
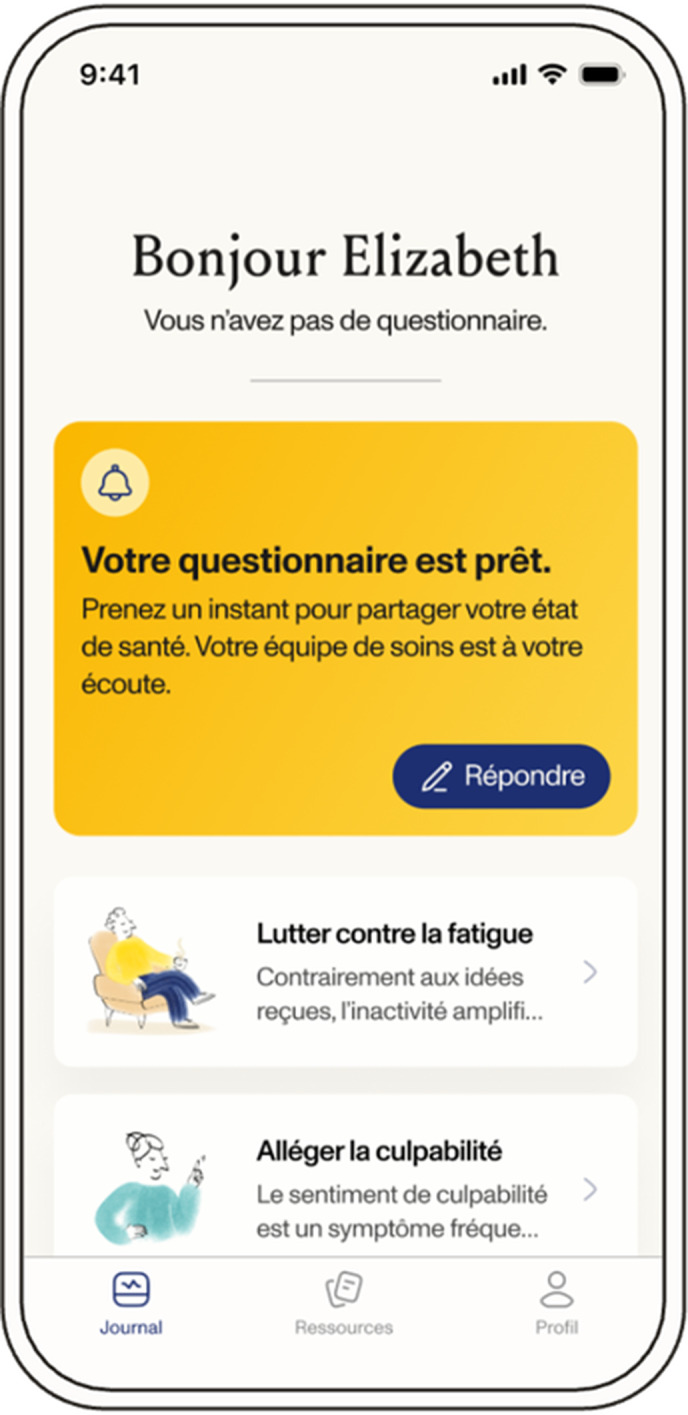
Image 2: Long description.

**Image 3:**
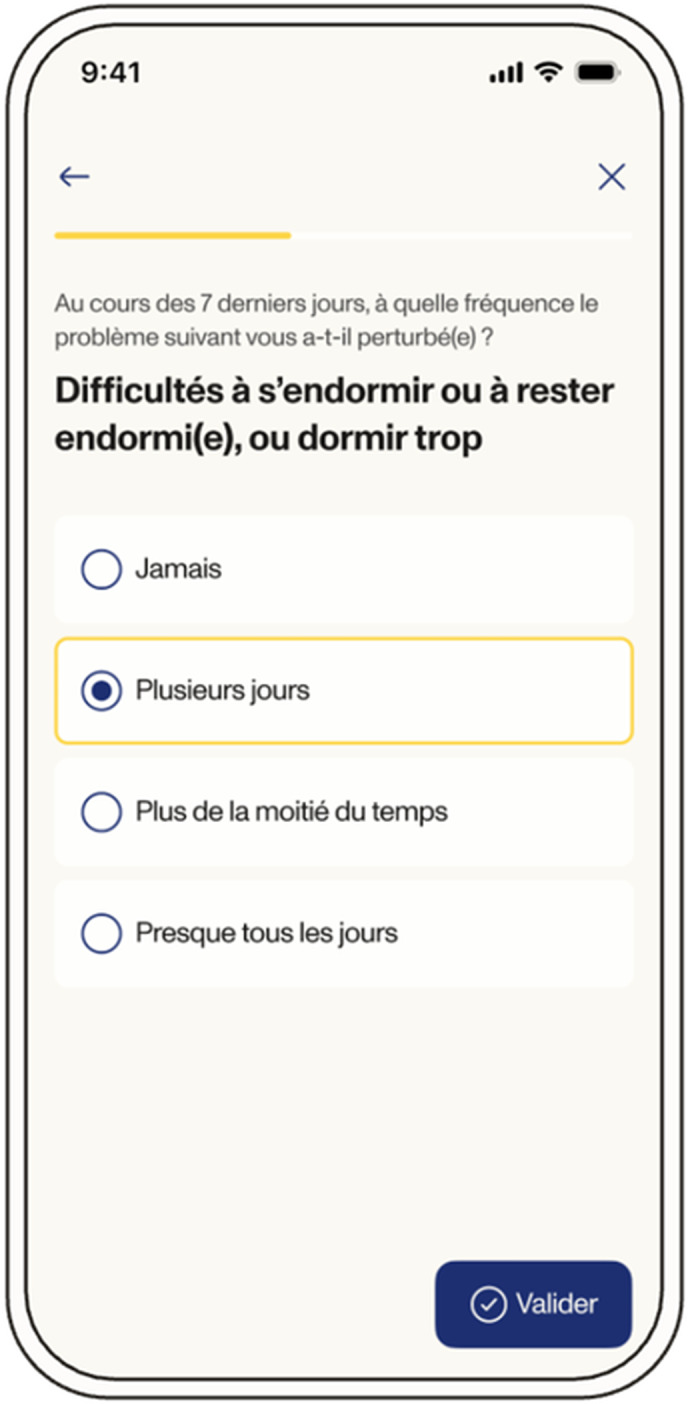

**Conclusions:**

The results will provide valuable insights into the clinical benefit of the RPM device Edra, particularly its impact on symptom improvement, patient adherence, care pathways, and overall quality of life in individuals with MDD.

**Disclosure of Interest:**

A. Yrondi: None Declared, A. Bourla: None Declared, R. Belzeaux: None Declared, E. Olié: None Declared, R. DAVID: None Declared, J. Mallet: None Declared, A. Amad: None Declared, A. Sauvaget: None Declared, M. Polosan: None Declared, J. Holtzmann: None Declared, F. Chevrier: None Declared, E. Haffen: None Declared, D. Bennabi: None Declared, F. Stéphan: None Declared, E. Artiges : None Declared, A. Amrandi-Chickh: None Declared, S. Bulteau : None Declared, W. El-Hage Consultant of: Air Liquide, Boehringer-Ingelheim, Chugai, Eisai, Janssen Cilag, Jazz Pharmaceuticals, Lundbeck, Mapreg SAS, Mindforce Game Lab, Novartis, Otsuka, UCB., V. Rousseau: None Declared, N. Lele : None Declared, C. Martelli: None Declared, L. Mekaoui: None Declared, D. Jacon : None Declared, G. Chabridon : None Declared, L. Dormegny Jeanjean: None Declared, F. Hermelin: None Declared, E. Touré Cuq: None Declared, P.-M. Llorca Consultant of: Ethypharm et Semeia

## Long descriptions

### Image 1: Long description

The interface is displayed on a laptop screen.

1. Left Sidebar: A narrow vertical navigation bar with icons for search, patients, notifications, and settings.

2. Patient Profile Column: Located on the left side of the main window. It lists administrative information for Elisabeth Desmaux, including birth date 01/1980 and gender Femme. Clinical information identifies the primary pathology as Bipolar Disorder F 31, diagnosed in 2020. The care team includes Dr. Fanny JACQ and three other members.

3. Monitoring Grid: Occupies the right two-thirds of the screen under the tab Télésurveillance.

* The horizontal axis lists dates from 7 Nov. to 15 Jan.

* The vertical axis lists health metrics including Treatment, Global Health E Q 5 D 5 L, Suicidal Ideation P H Q 9, Depression P H Q 9, Mania, and Anxiety G A D 7.

* Data points are represented by numerical values in a grid. Cells are color-coded from white (low/stable) to dark red (high/severe).

* Notable trends: Suicidal ideation and depression scores show a significant increase (dark red) starting from 26 Dec. through 15 Jan. Anxiety frequency and impact also show red intensity in the later dates.

* Adhesion and I M C (B M I) remain relatively stable at the bottom of the grid.

Navigate back to Image 1:.

### Image 2: Long description

The interface is displayed on a smartphone screen.

At the top, the status bar shows the time 9:41, signal strength, W i F i, and battery icons.

Below the status bar, a header reads Bonjour Elizabeth followed by the subtext Vous n'avez pas de questionnaire.

A prominent yellow notification card occupies the center. It features a bell icon in the top left and bold text stating Votre questionnaire est prêt. Below this, smaller text reads Prenez un instant pour partager votre état de santé. Votre équipe de soins est à votre écoute. A dark blue button in the bottom right of the card contains a pencil icon and the word Répondre.

Below the yellow card are two white list items with right-facing arrows.

1. The first item shows an illustration of a person sitting in a chair and is titled Lutter contre la fatigue with the snippet Contrairement aux idées reçues, l'inactivité amplifi...

2. The second item shows an illustration of a person's head and shoulders and is titled Alléger la culpabilité with the snippet Le sentiment de culpabilité est un symptôme fréque...

At the very bottom is a navigation bar with three icons and labels.

- Journal (selected, with a graph icon)

- Ressources (with a document icon)

- Profil (with a person icon)

Navigate back to Image 2:.

